# Aortic prosthetic graft thrombosis after bee sting-induced anaphylaxis

**DOI:** 10.1016/j.jvscit.2025.101990

**Published:** 2025-09-23

**Authors:** Elena-Mihaela Cordeanu, Solène Brouder, Corina Mirea, Camille Zamperini, Dominique Stephan

**Affiliations:** Department of Hypertension and Vascular Diseases, Clinical Pharmacology, Strasbourg University Hospitals, Strasbourg, France

**Keywords:** Hymenoptera envenomation, Anaphylaxis, Prosthetic graft thrombosis, Bee sting, Vascular surgery

## Abstract

Hymenoptera stings may cause severe cardiovascular events through direct prothrombotic venom effects and anaphylaxis-related hemodynamic collapse. We report the case of a 62-year-old male beekeeper with an infrarenal aortoaortic prosthetic graft who developed grade 3 anaphylaxis after a bee sting. Within 2 hours, he experienced bilateral lower limb paresthesia and severe pain. Computed tomography angiography revealed complete graft thrombosis extending into the left iliac axis. Emergency axillobifemoral bypass restored perfusion. Postoperative complications included compartment syndrome, acute kidney injury, and rhabdomyolysis, all resolving favorably. This case illustrates that hymenoptera venom can precipitate prosthetic graft occlusion through synergistic thrombogenic pathways, warranting preventive strategies and close monitoring in high-risk patients.

Hymenoptera, a large order of insects that includes bees, wasps, and hornets, are responsible for millions of stings annually, with clinical manifestations ranging from local cutaneous reactions to systemic anaphylaxis.^1^ Although systemic allergic responses are well recognized, arterial thrombosis represents a rare but severe complication. Cases of coronary artery occlusion, cerebral infarction, peripheral arterial thrombosis, and venous thrombosis have been documented after bee or wasp stings, typically attributed to the direct thrombogenic properties of venom components or to the hemodynamic instability during severe anaphylaxis.^2^, ^3^, ^4^, ^5^, ^6^ To date, thrombosis of prosthetic vascular grafts secondary to hymenoptera envenomation has not been described. We report the first case of complete infrarenal aortic prosthetic graft thrombosis secondary to bee sting-induced anaphylaxis, highlighting a previously unrecognized and potentially fatal complication in patients with vascular implants.

## Case presentation

A 62-year-old male beekeeper was stung on the left heel while tending his hives. His past medical history comprised an infrarenal aortic aneurysm treated in 2016 with an aortoaortic prosthetic graft. Additional comorbidities included cardiovascular risk factors (hypertension, active smoking, and age >50 years), heterozygous factor V Leiden mutation (FVL), and previous venous thromboembolic events (pulmonary embolism in 2013 and extensive left iliofemoral deep vein thrombosis in 2016). He had no history of claudication or aortoiliac occlusive disease, and regular vascular ultrasound follow-up confirmed the absence of iliac axis lesions before the event. The patient had a documented history of hymenoptera allergy but had not experienced severe systemic reactions previously. His regular medications included rivaroxaban 20 mg and ramipril/hydrochlorothiazide 5/12.5 mg daily. Rivaroxaban 20 mg had been taken at 8 am on the day of the event.

**Fig 1:**
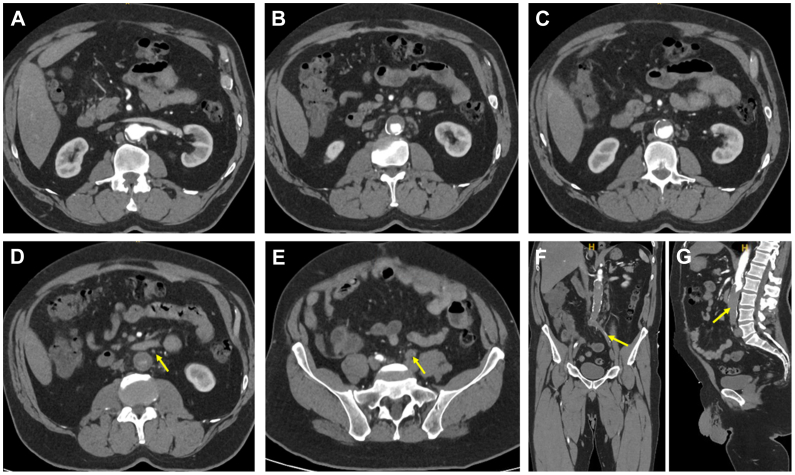
Contrast-enhanced computed tomography angiography showing complete thrombosis of the infrarenal aortic prosthetic graft. Sequential axial images **(A-E)** illustrate progressive craniocaudal occlusion from the suprarenal level to the pelvic vessels, with the complete absence of contrast enhancement within the graft. The inferior mesenteric artery (*yellow arrow*, **D**) and the left common iliac artery (*yellow arrow*, **E**) are occluded. Coronal **(F)** and sagittal **(G)** reconstructions depict patent suprarenal aorta with complete thrombotic obliteration of the infrarenal prosthetic segment extending into the left iliac axis (*yellow arrows*). Contrast enhancement is preserved in native vessels above the graft, with no distal perfusion below the occlusion.

Thirty minutes after sting, he developed grade 4 anaphylaxis with profound hypotension (systolic blood pressure 60 mm Hg), which persisted for approximately 25 minutes, profuse diarrhea, vomiting, severe abdominal pain, marked diaphoresis, lip angioedema, tachypnea, and diffuse erythema. Prehospital administration of prednisolone and chlorpheniramine stabilized his hemodynamics. Coagulation studies at admission revealed a prothrombin activity of 89% (reference: 70%-100%), International Normalized Ratio of 1.08, and an activated partial thromboplastin time within normal limits, consistent with therapeutic anticoagulation. Although no anti-Xa activity was measured on admission, the patient reported excellent adherence to rivaroxaban, with the last 20 mg dose taken at 8 am on the day of the event. Approximately 2 hours later, he reported bilateral lower limb paresthesia and progressive hypoesthesia accompanied by severe lumbar pain. Physical examination confirmed stage IIb acute limb ischemia. This clinical deterioration prompted immediate imaging with computed tomography angiography, which revealed a complete thrombosis of the infrarenal aortic prosthesis with extension into the left iliac axis, resulting in the complete absence of arterial flow to the left lower extremity and no reconstitution of the left hypogastric artery. On the right side, flow reconstituted at the level of the iliac bifurcation, and the right hypogastric artery was patent. The inferior mesenteric artery appeared occluded on imaging; however, details regarding its management during the original 2016 aortoaortic graft procedure were not available (Fig 1). The patient was transferred to a university vascular surgery center, and surgical revascularization started 4 hours after symptoms onset. Given the extent of thrombotic burden and bilateral lower limb ischemia, he underwent emergency axillobifemoral bypass surgery using the left axillary artery as inflow, with extensive thromboembolectomy of the left femoral arteries. Retrieved embolic material confirmed the thrombotic nature of the occlusion. Intraoperative arteriography demonstrated satisfactory graft patency and restoration of distal perfusion. The postoperative course was notable for left leg compartment syndrome requiring fasciotomy with negative-pressure wound therapy, rhabdomyolysis secondary to ischemia-reperfusion injury complicated by acute kidney injury with oliguria all resolving with supportive care.

Before hospital discharge, the patient recovered ambulation with crutches. Doppler ultrasound imaging confirmed bypass patency. Anticoagulation was switched from tinzaparin to apixaban after normalization of hepatic enzymes. Thrombophilia testing showed normal antithrombin and protein C, mild protein S deficiency (57%; normal >65%), negative lupus anticoagulant, and the absence of JAK2 V617 F mutation.

## Discussion

**Fig 2:**
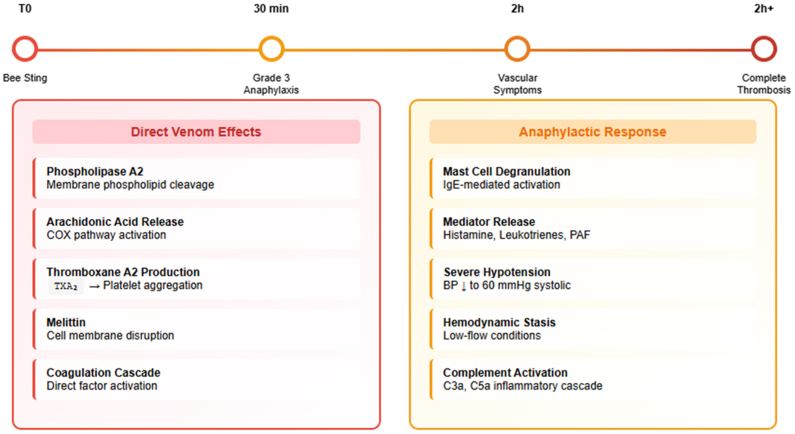
Pathophysiological timeline and dual mechanisms of prosthetic graft thrombosis after bee sting-induced anaphylaxis. The upper timeline illustrates the sequence from the initial bee sting (T0) to complete vascular thrombosis (>2 hours). Two parallel mechanisms converge to promote thrombosis. Left panel: Direct venom effects, with phospholipase A2-mediated arachidonic acid release leading to thromboxane A2 production and platelet aggregation, amplified by melittin-induced membrane disruption and direct activation of the coagulation cascade. Right panel: Anaphylactic response, involving mast cell degranulation, release of inflammatory mediators, severe hypotension (systolic blood pressure 60 mm Hg), and resultant hemodynamic stasis within the prosthetic graft. The synergy between direct thrombogenic venom components and anaphylaxis-induced low-flow conditions created optimal circumstances for complete prosthetic graft occlusion, despite therapeutic anticoagulation. *BP*, Blood pressure; *COX*, cyclooxygenase; *PAF*, platelet-activating factor; *TXA*_*2*_, thromboxane A2.

This case of complete aortic prosthetic graft thrombosis triggered by hymenoptera envenomation, highlights the convergence of multiple thrombogenic pathways that together created optimal conditions for this severe complication. Similar thrombotic phenomena have also been described in the setting of acute stent thrombosis after hymenoptera stings, further supporting the link between venom-induced procoagulant activity and thrombosis of cardiovascular implants.^7^ The underlying pathophysiology reflects a deleterious interaction between the direct procoagulant properties of bee venom and the profound hemodynamic disturbances induced by severe anaphylaxis, both acting on the inherently thrombogenic surface of a prosthetic vascular graft (Fig 2). Bee venom contains multiple bioactive compounds that directly activate thrombotic pathways, with phospholipase A2 serving as the primary thrombogenic mediator through its ability to cleave membrane phospholipids and release arachidonic acid, which is subsequently metabolized via the cyclooxygenase pathway to produce thromboxane A2, a potent platelet aggregator and vasoconstrictor that creates local conditions favoring clot formation. Melittin, another critical venom component, amplifies these effects by disrupting cellular membranes and further activating phospholipase A2, whereas hyaluronidase enhances venom penetration and systemic distribution, ensuring widespread exposure to these thrombogenic substances throughout the cardiovascular system.^8^ In addition, bee venom phospholipase A2 has been shown to activate the nuclear factor-κB signaling pathway, a central regulator of inflammatory and immune responses, which can upregulate tissue factor expression and thereby enhance procoagulant activity.^9^

The severe anaphylactic reaction experienced by our patient created a second, equally important thrombogenic mechanism through massive mast cell degranulation and the subsequent release of inflammatory mediators including histamine, leukotrienes, and platelet-activating factor, resulting in profound vasodilation, increased vascular permeability, and most critically for this case, severe hypotension with systolic pressure dropping to 60 mm Hg. The hemodynamic disturbance in this setting, characterized by low-flow stasis, is particularly detrimental for prosthetic vascular grafts, which lack the protective endothelial lining normally responsible for producing antithrombotic mediators such as nitric oxide, prostacyclin, and tissue plasminogen activator, making them highly susceptible to thrombus formation. Unlike native vessels endowed with intrinsic antithrombotic properties, prosthetic grafts possess nonendothelialized surfaces that inherently promote platelet activation and coagulation, while also favoring growth factor release and low-shear stress-mediated endothelial injury, processes that together drive intimal hyperplasia and make these grafts highly vulnerable to even minor disturbances in flow dynamics or hemostatic balance.^10^^,^^11^ The patient's prothrombotic background—heterozygous FVL mutation and previous venous thromboembolism—further amplified thrombotic risk transforming what might have been a manageable thrombotic stimulus into a severe occlusive arterial event. Beyond its well-established association with venous thromboembolism, the FVL mutation has also been linked to an increased risk of arterial thrombosis, particularly in the presence of additional prothrombotic or inflammatory triggers.^12^ FVL confers resistance to activated protein C, thereby prolonging the coagulation cascade and amplifying the tendency for clot formation in the presence of additional prothrombotic stimuli. Although the patient's heterozygous FVL mutation and the prolonged low-flow state due to anaphylactic shock were sufficient to predispose to thrombosis, the close temporal relationship with the bee sting and the established thrombogenic properties of venom strongly suggest a synergistic mechanism. The sequence of events, with initial severe anaphylaxis followed by delayed ischemia, is consistent with venom-induced platelet activation initiating thrombus formation, while sustained hypotension propagated complete graft occlusion. Histological examination of the retrieved specimen during surgical thromboembolectomy demonstrated fibrino-cruoric thrombus with scattered inflammatory cells and no evidence of advanced organization, consistent with an acute thrombotic process that overwhelmed the patient's therapeutic anticoagulation with rivaroxaban. Although pre-existing aortoiliac disease could have contributed to stasis, the absence of claudication and the lack of significant iliac stenosis on prior ultrasound imaging and computed tomography angiography argue against this explanation. Unlike native vessels endowed with intrinsic antithrombotic properties, prosthetic grafts possess nonendothelialized surfaces that inherently promote platelet activation and coagulation, making them highly vulnerable to even minor disturbances in flow dynamics or hemostatic balance.^13^ The failure of rivaroxaban to prevent graft thrombosis in this case warrants specific discussion, as it highlights the limitations of direct-acting oral anticoagulants alone in managing platelet-driven thrombosis on prosthetic surfaces. Although rivaroxaban effectively prevents cardioembolic events through factor Xa inhibition, it demonstrates limited efficacy against platelet-rich thrombi that predominate on prosthetic surfaces lacking protective endothelial mediators such as nitric oxide, prostacyclin, and tissue plasminogen activator.^14^ Furthermore, in vitro studies have shown that rivaroxaban does not modify platelet aggregation or adhesion on collagen fibers at therapeutic concentrations.^15^ The venom-induced massive platelet activation, combined with anaphylaxis-related hemodynamic stasis, created conditions where the anticoagulant effect of rivaroxaban was insufficient to prevent thrombosis. This phenomenon parallels the documented limitations of direct oral anticoagulants in mechanical heart valve thrombosis, where platelet activation mechanisms predominate over coagulation cascade activation.^16^ This case underscores that prosthetic vascular grafts are uniquely vulnerable to combined thrombogenic insults and that patients with both vascular implants and hymenoptera allergies require heightened preventive and postexposure monitoring strategies.

## Conclusions

Hymenoptera envenomation can trigger catastrophic vascular graft thrombosis through synergistic mechanisms combining direct venom-induced hypercoagulability via phospholipase A2 and thromboxane A2 production and anaphylaxis-induced hemodynamic compromise. The lack of endothelial protection in prosthetic grafts greatly amplifies this risk significantly. Patients with vascular prostheses and known hymenoptera allergy should be counseled on preventive measures, including prompt use of adrenaline autoinjectors, and undergo enhanced postexposure vascular monitoring even in the absence of early ischemic symptoms given the potential for catastrophic thrombotic complications. In cases of severe anaphylaxis with prolonged hemodynamic instability, consideration should be given to intensified anticoagulation monitoring and potential temporary parenteral anticoagulation, weighing thrombotic risk against bleeding complications on a case-by-case basis.

## Patient consent

Informed consent was obtained from the patient for publication.

## Funding

None.

## Declaration OF generative AI and AI-assisted technologies in the writing process

During the preparation of this work the author(s) used ChatGPT (GPT-5, OpenAI) and Claude (Sonnet 4, Anthropic) in order to assist with language formulation and translation. After using this tool/service, the author(s) reviewed and edited the content as needed and take(s) full responsibility for the content of the publication.

## Disclosures

None.
